# MiR-24 functions as a tumor suppressor in nasopharyngeal carcinoma through targeting *FSCN1*

**DOI:** 10.1186/s13046-015-0242-6

**Published:** 2015-10-26

**Authors:** Ying-Qing Li, Jian-Hua Lu, Xue-Ming Bao, Xi-Fu Wang, Jun-Hua Wu, Wei-Qiang Hong

**Affiliations:** Guangzhou First People’s hospital, Guangzhou Medical University, 1 Panfu Road, Guangzhou, Guangdong 510180 PR China

**Keywords:** Nasopharyngeal carcinoma, miR-24, Fascin homologue 1, Cell growth, Invasion, Metastasis

## Abstract

**Background:**

Increasing evidence indicates that the dysregulation of miRNAs expression is involved in the tumorigenesis by acting as tumor suppressors or oncogenes. However, no study investigates the function and mechanisms of miR-24 in nasopharyngeal carcinoma (NPC).

**Methods:**

Quantitative RT-PCR, MTT, colony formation, soft-agar, wound healing, Transwell migration and invasion assays, and xenograft tumor growth and lung metastasis models were performed to test the expression levels and functions of miR-24 in NPC. Luciferase reporter assay, quantitative RT-PCR, Western blotting, and immunohistochemistry were used to identify and verify the target of miR-24.

**Results:**

The results showed that MiR-24 was obviously downregulated in NPC cell lines and tissue samples (*P* < 0.05). Ectopic expression of miR-24 inhibited the cell viability, proliferation, migration, and invasion in vitro (all *P* < 0.05), and suppressed the xenograft tumor growth and lung metastasis formation in vivo (all *P* < 0.05). Fascin homologue 1 (*FSCN1*) was verified as a direct target of miR-24, and silencing *FSCN1* expression with small interfering RNA inhibited NPC cell proliferation and invasion (all *P* < 0.05).

**Conclusions:**

Overall, miR-24 acts as a novel tumor suppressor in the development and progression of NPC through targeting *FSCN1*, which providing new insight into the mechanisms of NPC carcinogenesis and suggesting the possibility of miR-24 as a therapeutic target.

## Introduction

Nasopharyngeal carcinoma (NPC) is an epithelial malignancy located in the nasopharynx. It is common in Southern China and Southeast Asia, while it is rare in most of the other regions of the world. The age-standardized incidence ranges from 20-50 per 100,000 males in Southern China to 0.5 in Western Countries. The data from the International Agency for Research on Cancer showed that approximately 84,000 new cases of NPC and 51,600 deaths were reported worldwide in 2008 [1]. Although considerable advances made in multimodal treatment, the prognosis of NPC patients, especially with locoregionally advanced disease, remains very poor due to the recurrence and/or distant metastasis [2, 3]. Therefore, a better understanding of the molecular mechanisms, involving in the carcinogenesis and progression of NPC, is critical for the development of novel treatment strategies for NPC patients.

MiRNAs are small noncoding, double-stranded RNA molecules that can regulate protein expression at the post-transcriptional level by base pairing to the 3’ untranslated region (UTR) of their target genes based on complete or incomplete sequence complementarity [4, 5]. As we known, a single miRNA can modulate several target genes and one gene may be modulated by several miRNAs, underlying the formation of complex regulatory networks [3]. MiRNAs have been demonstrated to be involved in diverse biological processes [6, 7], and the dysregulation of miRNA expression has been found to be associated with carcinogenesis [8–10]. Recently, it has been reported that human miRNAs are frequently located at the fragile sites and genomic regions that involved in cancers, and miRNAs may function as tumor suppressors or oncogenes [11–13]. Dysregulated expression of miRNAs has been found in most types of human cancers [8–10], including NPC [14–16]. Recent studies have also reported that several downregulated or overexpressed miRNAs, such as miR-26a, miR-29c, miR-34c, miR-93, and miR-633, involve in NPC development and progression by modulating cell growth, proliferation, apoptosis, and invasion [17–21]. These findings indicate that the dysregulated expression of miRNAs plays important roles in NPC carcinogenesis, therefore, more comprehensive investigation is needed to fully clarify the function of miRNAs in NPC development and progression.

In this study, we investigated the biological function and molecular mechanism of miR-24 in NPC. We found that miR-24 was decreased in NPC cell lines and clinical specimens. We further demonstrated that overexpression of miR-24 suppressed the NPC cell proliferation and migration in vitro, and inhibited the xenograft tumor growth and lung metastasis in vivo. Moreover, we identified and verified that fascin homologue 1 (*FSCN1*) was a functional target of miR-24. Our findings elucidate the roles of miRNAs in NPC and further contribute to develop novel therapeutic strategies for NPC.

## Materials and methods

### Cell lines and tissue samples

Six human NPC cell lines (CNE-1, CNE-2, C666-1, SUNE-1, HNE-1, and HONE-1) were grown in RPMI-1640 (Invitrogen, Carlsbad, CA, USA) supplemented with 10 % FBS (Gibco, Grand Island, NY, USA). The immortalized nasopharyngeal epithelial cell line NP69 was cultured in Keratinocyte/serum-free medium (Invitrogen) supplemented with bovine pituitary extract (BD Biosciences, San Jose, CA, USA). 293FT cells were maintained in DMEM (Invitrogen) supplemented with 10 % FBS. Eighten freshly-frozen NPC and nine normal nasopharyngeal epithelium tissue samples were obtained from Guangzhou First People’s hospital. This study was approved by the institutional ethical committee of Guangzhou First People's hospital and written informed consent was obtained from each patient.

### RNA extraction and reverse transcription

Total RNA was extracted by TRIzol reagent (Invitrogen) according to the manufacturer’s instructions. 2 μg of total RNA was reverse-transcribed using M-MLV reverse transcriptase (Promega, Madison, WI, USA) and Bulge-Loop™ specific RT-primers (RiboBio, Guangzhou, China) for miRNA or random primers (Promega) for mRNA. The reverse-transcription was performed by incubation at 42 °C for 60 min and then at 70 °C for 10 min in a 50 μl reaction volume for miRNA or incubation at 37 °C for 60 min and then at 70 °C for 15 min in a 20 μl reaction volume for mRNA. All reverse transcription product was stored at -20 °C until use.

### Quantitative PCR

The PCR reactions were done on a Bio-Rad CFX96 sequence detection system (Bio-Rad Laboratories Inc., Hercules, CA, USA) by pre-incubating at 95 °C for 2 min, followed by 40 cycles of denaturation at 95 °C for 15 s and annealing/extension at 60 °C for 30 s in a 20 μl reaction volume containing 2 μl reverse transcription product, 9 μl SYBR Green qPCR SuperMix-UDG reagents (Invitrogen), 4 μl PCR forward and reverse primer mix, and 5 μl H_2_O_2_. Primers for miR-24 and U6 amplification were purchased from RiboBio. Primers for *FSCN1* amplification were listed as follows: 5′-CTACAACATCAAAGACTCCACAG-3 (forward) and 5′-ATGGCCACCTTGTTATAGTC-3′ (reverse); and primers for GAPDH amplification were: 5′-CTCCTCCTGTTCGACAGTCAGC-3′ (forward) and 5′-CCCAATACGACCAAATCCGTT-3′ (reverse). All of the reactions were performed in triplicate. The U6 or GAPDH were used as normalized controls, and the relative levels were calculated using the 2^-ΔΔCT^ equation.

### Transient transfection and generation of stably transfected cell lines

The miR-24 mimics (miR-24), miRNA control (miR-Ctrl), small interfering RNA for *FSCN1* (siFSCN1), and siRNA control (siNC) were purchased from GenePharma (GenePharma, Suzhou, China). After seeded into 6-well plates, cells were transfected with miRNA mimics or siRNA at a final concentration of 50 or 100 nM respectively using Lipofactamine 2000 Reagent (Invitrogen). The genomic region that included pri-miR-24 was cloned into pMSCV-puromycin vector. A plasmid mixture containing retroviral packaging PIK vector and pMSCV-miR-24 or empty pMSCV vector were co-transfected into 293FT cells. After transfection, we harvested cell supernatants and infected SUNE-1 cells, and further selected the stably transfected cells with puromycin.

### MTT, colony formation and soft-agar assays

For MTT assay, cells were seeded into 96-well plates (1500 cells/well) after transfection, and the cell viability was detected at 1, 2, 3, 4, and 5 days by measurement of the absorbance at 490 nm using a spectrophotometric plate reader. For colony formation assay, cells were seeded into 6-well plates (500 cells/well) after transfection, and the colonies were fixed, stained, and counted after culture for 7 or 12 days. For soft-agar assay, cells (2.5 × 10^4^) were resuspended in 1 ml of complete medium containing 0.66 % agar (Sigma, St. Louis, MO, USA) and then added to the top of a 1 % agar/complete medium layer in six-well plates. After culture for 9 or 12 days, the colonies were counted under an inverted microscope.

### Wound healing, transwell migration and invasion assays

For wound healing assay, cells were seeded into 6-well plates after transfection, and the artificial wounds were created by scraping the cell monolayer with a sterile micropipette tip. Representative images of cells migrating into wounds were captured at 0 and 24 h. Migration and invasion assays were performed using Transwell chambers with 8 μm pore polycarbonate membrane insert (Corning, Lowell, MA, USA) coated without or with Matrigel (BD Biosciences). 5 × 10^4^ or 1 × 10^5^ cells in serum-free medium were placed into the upper chamber for migration or invasion assay, and medium supplementary with 10 % FBS was added to the lower chamber. After 10 h or 24 h, the migrated or invaded cells were fixed, stained, and counted.

### Tumor xenograft and lung metastasis models

Male BALB/c nude mice (4 ~ 5 weeks old) were purchased from the Medical Experimental Animal Center of Guangdong Province (Guangzhou, China). All protocols were approved by the Animal Care and Use Ethnic Committee. 1 × 10^6^ SUNE-1 cells stably overexpressing miR-24 or scramble miRNA were subcutaneously injected in the dorsal flank or intravenously injected through the tail vein. For tumor xenograft assay (*n* = 5 for each group), tumor size was measured every three days, and the tumor volumes were calculated. Mice were sacrificed, and tumors were dissected and weighted after 30 days. For lung metastasis assay (*n* = 5 for each group), mice were sacrificed, the lungs were fixed, paraffin-embedded, cut, and stained with H&E staining after eight weeks.

### Luciferase reporter assay

The wild-type (Wt) FSCN1-3′UTR and mutant FSCN1-3′UTR containing the putative binding site of miR-24 were established and cloned in the Firefly luciferase-expressing vector psiCHECK™ (Promega). Cells were seeded into 24-well plates the day before transfection, and transfected with either the psiCHECK-FSCN1-Wt or the psiCHECK-FSCN1-Mt reporter vector, together with the Renilla luciferase-expressing vector pRL-TK (Promega) and miR-24 mimics or miR-Ctrl using Lipofectamine 2000 (Invitrogen). After 48 h, cells were harvested, and luciferase activities were detected with the Dual-Luciferase Reporter System (Promega).

### Western blotting

Total protein was extracted using RIPA buffer with protease inhibitor Cocktail (Pierce, Rockford, IL, USA), and then quantified with the BCA protein Assay Kit (Pierce). Protein was separated by 9 % SDS-PAGE, and then transferred onto PVDF membranes (Millipore, Bedford, MA, USA). Then, the membranes were incubated by rabbit monoclonal anti-FSCN1 antibody (1:5000; Epitomics, Burlingame, CA, USA) or mouse monoclonal anti-GAPDH antibody (1:5000, Abcam, Cambridge, MA, USA) followed by incubation with secondary antibody (Sigma). Signals were determined with an enhanced chemiluminescence detection system.

### Immunohistochemistry

After deparaffinization, rehydration, antigenic retrieval, and quenching the endogenous peroxidase activity, the sections for immunohistochemical staining were incubated with rabbit monoclonal anti-FSCN1 antibody (1:200; Epitomics) followed by secondary antibody bound to HRP-conjugated streptavidin. The bound antibody was visualized by 3,3-diaminobenzidine, and sections were counterstained. The staining scores (0, 1, 2, 3, 4, 6, 8, 9, 12) for FSCN1 expression was calculated as the product of the proportion of positively stained tumor cells (1, <10 %; 2, 10-35 %; 3, 36-75 %; 4, >75 %) and the intensity of staining (0, no staining; 1, weak staining, light yellow; 2, moderate staining, yellow brown; 3, strong staining, brown).

### Statistical analysis

Data were presented as mean ± SD, and differences between groups were analyzed using the Student’s *t*-test. All statistical analysis was performed with the SPSS 16.0 software, and two tailed *P* values of < 0.05 were defined as statistically significant.

## Results

### MiR-24 is downregulated in NPC cell lines and tissue samples

**Fig. 1:**
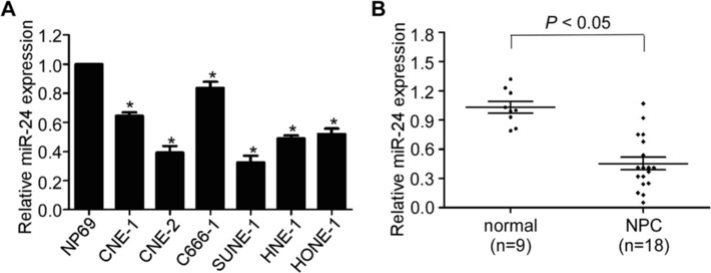
MiR-24 is downregulated in NPC cell lines and tissue samples. **a** Relative expression of miR-24 in six NPC cell lines and NP69. **b** Relative expression of miR-24 in 18 freshly-frozen NPC and 9 normal nasopharyngeal epithelial tissue samples. U6 was used as normalized control. Data is presented as the mean ± SD; *P* values were calculated with the Student’s *t*-test

To explore the expression levels of miR-24 in NPC, quantitative RT-PCR were conducted in six NPC cell lines and an immortalized nasopharyngeal epithelial cell line NP69. Expression levels of miR-24 were downregulated in all NPC cell lines compared with NP69 (Fig. 1a). Expression levels of miR-24 were further detected in 18 freshly-frozen NPC and nine normal nasopharyngeal epithelial tissue samples. The expression of miR-24 was remarkably decreased in NPC tissues compared to normal tissues (Fig. 1b, *P* < 0.05).

### MiR-24 inhibits NPC cell growth and proliferation in vitro

**Fig. 2:**
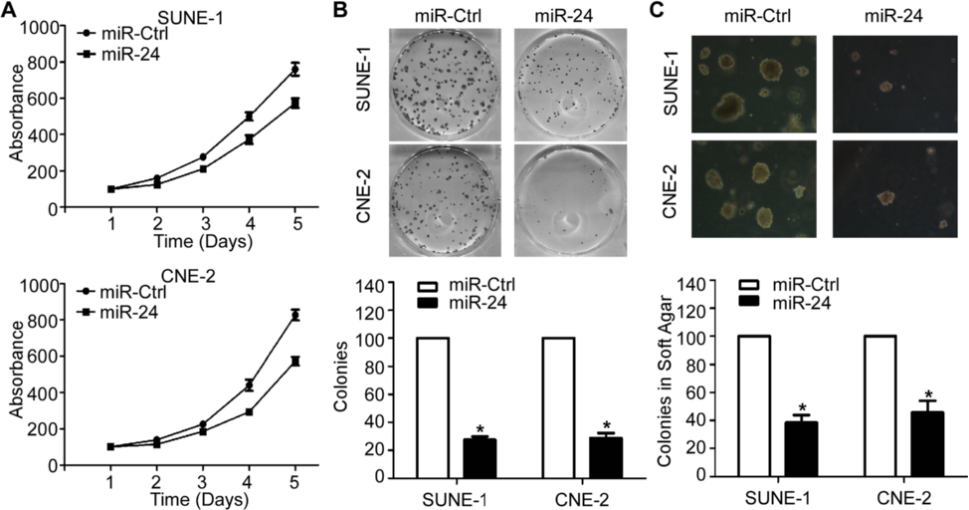
MiR-24 inhibits NPC cell growth and proliferation in vitro. **a** Cell viability detected by MTT assay. **b** Cell anchorage-dependent proliferation detected by colony formation assay. **c** Cell anchorage-independent proliferation detected by soft-agar assay. Data is presented as the mean ± SD; *P* values were calculated with the Student’s *t*-test

To investigate the biological function of miR-24 in the development and progression of NPC, we performed MTT, colony formation, and soft-gar assays after transient transfection NPC cells with miR-24 mimics or miR controls. As shown in Fig. 2a, SUNE-1 and CNE-2 cells transfected with miR-24 mimics displayed a significant growth inhibition compared to those transfected with miR control (*P* < 0.05). Moreover, cells transfected with miR-24 mimics formed fewer and smaller colonies compared with cells transfected with miR controls in both anchorage-dependent and anchorage-independent manners (Fig. 2b-c, *P* < 0.05).

### MiR-24 inhibits NPC cell migration and invasion in vitro

**Fig. 3:**
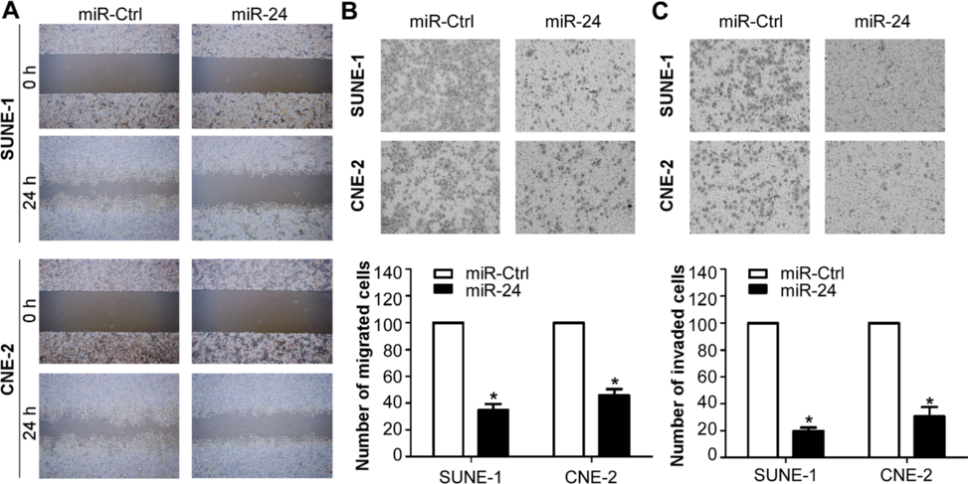
MiR-24 inhibits NPC cell migration and invasion in vitro. **a** Cell migratory ability detected by wound healing assay. **b** Cell migratory ability detected by Transwell migration assay. **c** Cell invasive ability detected by Transwell invasion assay. Data is presented as the mean ± SD; *P* values were calculated with the Student’s *t*-test

We further evaluated the effect of miR-24 on the migratory and invasive ability of NPC cells after transient transfection with miR-24 mimics or miR controls. The wound healing assay showed that the migratory ability of SUNE-1 and CNE-2 cells transfected with miR-24 mimics was much weaker than those transfected with miR controls (Fig. 3a). Transwell migration assay also demonstrated that overexpression of miR-24 remarkably suppressed the migratory ability of NPC cells (Fig. 3b, *P* < 0.05). In addition, Transwell invasion assay indicated that the invasive ability was inhibited by transfecting with miR-24 mimics (Fig. 3c, *P* < 0.05).

### MiR-24 inhibits NPC xenograft tumor growth and lung metastasis in vivo

**Fig. 4:**
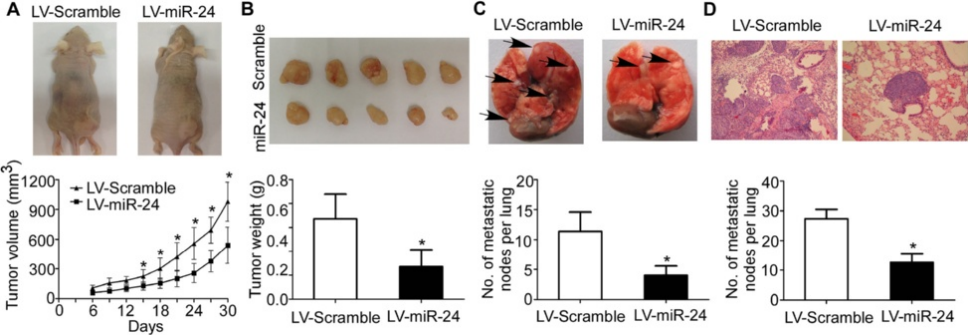
MiR-24 inhibits NPC xenograft tumor growth and lung metastasis in vivo. **a** Representative images of tumors formed and the growth curves of tumor volume. **b** Representative images of tumors formed and the tumor weight. **c** Representative images and quantification of macroscopic lung metastasis. **d** Representative images and quantification of microscopic lung metastatic nodes based on H&E staining. Data is presented as the mean ± SD; *P* values were calculated with the Student’s *t*-test

Given that miR-24 inhibited the proliferation and invasion of NPC cells in vitro, we tested whether ectopic expression of miR-24 could affect the tumorigenesis and metastasis in vivo. Xenograft tumor growth and lung metastasis models were obtained as described in the methods section. As shown in Fig. 4a, tumors grew at a slower rate and had smaller volumes in the miR-24 overexpressing group than the control group (*P* < 0.05). The average tumor weight in the miR-24 overexpressing group was also significantly lower (0.27 ± 0.14 g vs. 0.67 ± 0.20 g; Fig. 4b, *P* < 0.05). In addition, fewer metastatic nodes were formed on the surface of lungs in the miR-24 overexpressing group than the control group (Fig. 4c, *P* < 0.05), which was further confirmed by H&E staining (Fig. 4d, *P* < 0.05).

### FSCN1 is verified as a direct target of miR-24

**Fig. 5:**
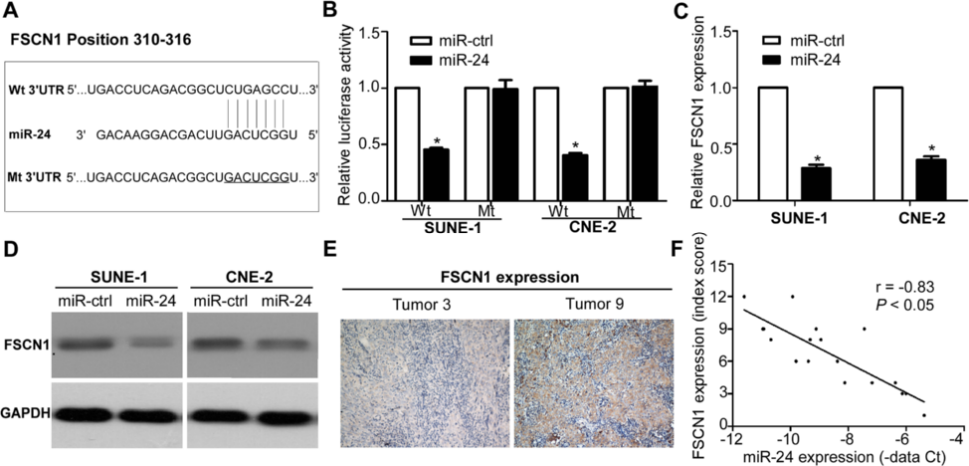
*FSCN1* is verified as a direct target of miR-24. **a** Wild-type and mutant miR-24 target sequences of *FSCN1* 3’UTR. **b** Relative luciferase activity detected by Luciferase reporter assay. **c** Quantification of *FSCN1* mRNA expression by quantitative RT-PCR. **d** *FSCN1* protein expression by Western blotting. **e** Representative images of *FSCN1* protein expression detected by immunohistochemistry. **f** Spearman’s correlation analysis of miR-24 and *FSCN1* protein expression. Data is presented as the mean ± SD; *P* values were calculated with the Student’s *t*-test

To address the molecular mechanism of miR-24 in NPC, we searched for candidate target genes by bioinformatics with publicly available databases (TargetScan and miRBase). *FSCN1* was identified as a target as it has one potential miR-24 binding site within its 3’ UTR (Fig. 5a). Luciferase reporter assay verified that *FSCN1* was a direct target of miR-24 because ectopic expression of miR-24 could inhibit the luciferase activity of the psiCHECK-FSCN1-Wt reporter vector but not the psiCHECK-FSCN1-Wt (Fig. 5b, *P* < 0.05). Furthermore, ectopic expression of miR-24 could suppress *FSCN1* expression at both the mRNA and protein levels (Fig. 5c-d, *P* < 0.05), and the expression of miR-24 was inversely correlated with *FSCN1* protein expression in clinical samples (Fig. 5e-f, *P* < 0.05).

### FSCN1 is involved in NPC cell proliferation and invasion

**Fig. 6:**
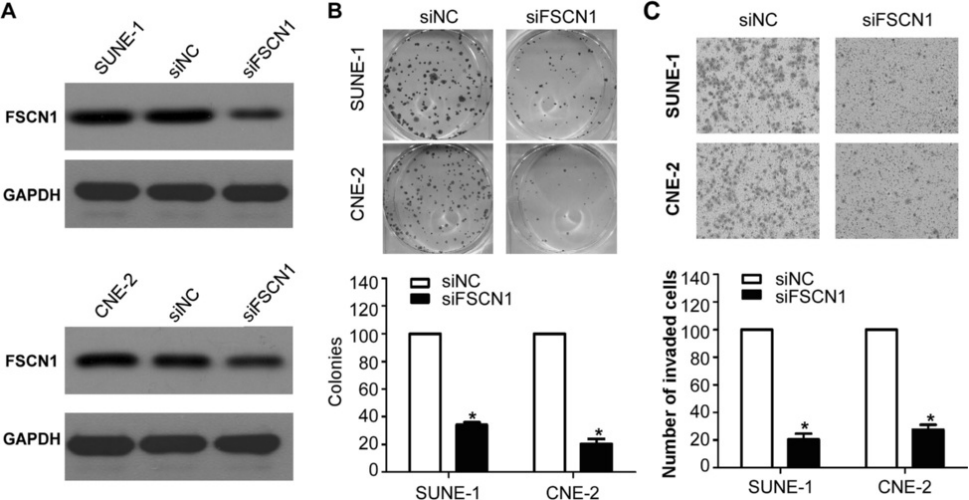
*FSCN1* is involved in NPC cell growth and invasion. **a** *FSCN1* protein expression by Western blotting. **b** Cell proliferation detected by colony formation assay. **c** Cell invasive ability detected by Transwell invasion assay. Data is presented as the mean ± SD; *P* values were calculated with the Student’s *t*-test

Finally, to determine whether *FSCN1* could affect the proliferation and invasion of NPC cells, we performed colony formation and Transwell invasion assays after transient transfection SUNE-1 and CNE-2 cells with siFSCN1 or siRNA control. As shown in Fig. 6a, Western blotting confirmed that siFSCN1 obviously suppressed the protein expression of *FSCN1*. Cells transfected with siFSCN1 formed fewer and smaller colonies than cells transfected with siRNA control (Fig. 6b, *P* < 0.05). In addition, the invasive ability of SUNE-1 and CNE-2 cells was significantly inhibited by transfecting siFSCN1 (Fig. 6c, *P* < 0.05).

## Discussion

Increasing evidence indicates that the dysregulation of miRNA expression is involved in the tumorigenesis of human cancers by functioning as tumor suppressors or oncogenes [8–13]. In our present study, we found that miR-24 was obviously downregulated in NPC cell lines and tissue samples. Ectopic overexpression of miR-24 inhibited cell viability, proliferation, migration, and invasion in vitro, and inhibited xenograft tumor growth and lung metastasis in vivo. Furthermore, *FSCN1* was verified as a direct target of miR-24, and involved in NPC cell proliferation and invasion. Taken together, our results suggest that miR-24 functions as a tumor suppressor by inhibiting NPC cell proliferation and invasion through targeting the oncogene of *FSCN1* and contribute to the development and progression of NPC.

Recently, several studies have reported that miRNAs were dysregulated in NPC tissues by miRNA expression profiling [14–17], and the dysregulation of specific miRNAs involved in NPC proliferation, apoptosis, invasion, and metastasis [17–21]. Lu et al. reported that miR-26a could suppress NPC cell growth and tumorigenesis by targeting enhancer of zeste homolog 2 (*EZH2*) [17]. Liu et al. found that miR-29c could suppress NPC cell invasion and metastasis by targeting T cell lymphoma invasion and metastasis 1 (*TIAM1*) [18]. Li et al. reported that miR-34c could inhibit NPC tumor growth and metastasis by targeting *MET* [19]. Xu et al. reported that miR-93 could promote NPC cell growth and invasion via targeting Disabled homolog-2 (*Dab-2*) [20]. Yi et al. found that miR-663 could promote NPC proliferation and tumorigenesis by targeting cyclin-dependent kinase inhibitor 1A (*p21*) [21]. Taken together, these findings indicate that the dysregulated miRNA expression plays important roles in NPC carcinogenesis; therefore, more comprehensive investigation is needed to fully clarify the function of miRNAs in NPC development and progression.

MiR-24 regulates tumor cellular function by acting as a tumor suppressor or oncogene in a cell type-specific manner [22–25]. It has also been reported that miR-24 could serve as diagnostic or prognostic biomarkers in several cancers [26–28]. In acute myeloid leukemia, miR-24 was firstly found as an oncogene by stimulating myeloid cell growth and proliferation via repression of the mitogen-activated protein kinase phosphatase 7 (*MKP-7*) [22]. MiR-24 was also demonstrated as an oncogene in glioma by promoting proliferation and invasion via targeting the suppression of tumorigenicity 7-like (*ST7L*) [23]. In contrast, miR-24 was demonstrated to act as a tumor suppressor in colon cancer cells by targeting dihydrofolate reductase (*DHFR*) [24]. Duan et al. recently also found that miR-24 functions as a tumor suppressor in gastric cancer by downregulating regenerating islet-derived family, member 4 (*RegIV*) [25]. In this study, miR-24 was found to be obviously decreased in NPC cell lines and tissue samples. Ectopic overexpression of miR-24 suppressed NPC cell viability, proliferation, migration, and invasion in vitro, and inhibited xenograft tumor growth and lung metastasis in vivo. Similarly, Wang et al. reported that miR-24 was reduced in the recurrent NPC tissues compared with the primary NPC tissues, and overexpression of miR-24 also inhibited NPC cell growth in vitro and reduced tumor growth in mouse models, and miR-24 is a potential radiosensitizor in NPC [29]. These findings suggest that miR-24 functions as a tumor suppressor in the tumorigenesis and progression of NPC.

Each individual miRNA can target several genes that own target sequence in their 3’ UTR and complement to the seed region of the miRNA. Several target genes of miR-24 have been identified and verified, such as *MKP-7*, *ST7L*, *DHFR*, *RegIV*, *BCL2* (B cell lymphoma 2), and *Net1* (nucleotide exchange factor 1) [22–25, 30, 31]. In our present report, we confirmed *FSCN1* as a novel direct target of miR-24 using luciferase reporter gene assay. Furthermore, overexpression of miR-24 could suppress the mRNA and protein expression of *FSCN1* and miR-24 expression was inversely correlated with the *FSCN1* protein expression. Several studies have reported that *FSCN1* is overexpressed in malignant tumors, and is generally correlated with its aggressive behavior by increasing cell motility and tumor invasiveness [32–34]. It has also been reported that *FSCN1* was upregulated in NPC and its overexpression was associated with poor survival and correlated with invasion [35]. In this study, we further found that inhibiting *FSCN1* expression with small interfering RNA remarkably suppressed cell proliferation and invasion. These results suggest that miR-24 inhibit cell growth and invasion by targeting *FSCN1*.

In this study, we found that miR-24 was obviously downregulated in NPC cell lines and tissue samples, and ectopic expression of miR-24 could suppress NPC cell growth and invasion through targeting *FSCN1*. MiR-24 functions as a novel tumor suppressor in the development and progression of NPC, proving new insight into the mechanisms of NPC carcinogenesis, and suggesting the possibility of miR-24 as a therapeutic target for NPC.

## References

[1] Jemal A, Bray F, Center MM, Ferlay J, Ward E, Forman D (2011). Global cancer statistics. CA Cancer J Clin.

[2] Rottey S, Madani I, Deron P, Van Belle S (2011). Modern treatment for nasopharyngeal carcinoma: current status and prospects. Curr Opin Oncol.

[3] Lin S, Guo Q, Wen J, Li C, Lin J, Cui X (2014). Survival analyses correlate stanniocalcin 2 overexpression to poor prognosis of nasopharyngeal carcinoma. J Exp Clin Cancer Res.

[4] Bartel DP (2004). MicroRNAs: genomics, biogenesis, mechanism, and function. Cell.

[5] He L, Hannon GJ (2004). MicroRNAs: small RNAs with a big role in gene regulation. Nat Rev Genet.

[6] Ambros V (2003). MicroRNA pathways in flies and worms: growth, death, fat, stress, and timing. Cell.

[7] Ambros V (2004). The functions of animal microRNAs. Nature.

[8] Calin GA, Croce CM (2006). MicroRNA signatures in human cancers. Nat Rev Cancer.

[9] Calin GA, Croce CM (2006). MicroRNA-cancer connection: the beginning of a new tale. Cancer Res.

[10] Lu J, Getz G, Miska EA, Alvarez-Saavedra E, Lamb J, Peck D (2005). MicroRNA expression profiles classify human cancers. Nature.

[11] Calin GA, Sevignani C, Dumitru CD, Hyslop T, Noch E, Yendamuri S (2004). Human microRNA genes are frequently located at fragile sites and genomic regions involved in cancers. Proc Natl Acad Sci U S A.

[12] Esquela-Kerscher A, Slack FJ (2006). Oncomirs - microRNAs with a role in cancer. Nat Rev Cancer.

[13] He L, Thomson JM, Hemann MT, Hernando-Monge E, Mu D, Goodson S (2005). A microRNA polycistron as a potential human oncogene. Nature.

[14] Liu N, Chen NY, Cui RX, Li WF, Li Y, Wei RR (2012). Prognostic value of a microRNA signature in nasopharyngeal carcinoma: a microRNA expression analysis. Lancet Oncol.

[15] Chen HC, Chen GH, Chen YH, Liao WL, Liu CY, Chang KP (2009). MicroRNA deregulation and pathway alterations in nasopharyngeal carcinoma. Br J Cancer.

[16] Sengupta S, den Boon JA, Chen IH, Newton MA, Stanhope SA, Cheng YJ (2008). MicroRNA 29c is down-regulated in nasopharyngeal carcinomas, up-regulating mRNAs encoding extracellular matrix proteins. Proc Natl Acad Sci U S A.

[17] Lu J, He ML, Wang L, Chen Y, Liu X, Dong Q (2011). MiR-26a inhibits cell growth and tumorigenesis of nasopharyngeal carcinoma through repression of EZH2. Cancer Res.

[18] Liu N, Tang LL, Sun Y, Cui RX, Wang HY, Huang BJ, et al. MiR-29c suppresses invasion and metastasis by targeting TIAM1 in nasopharyngeal carcinoma. Cancer Lett. 2013;329:181–8. 10.1016/j.canlet.2012.10.032 23142282

[19] Li YQ, Ren XY, He QM, Xu YF, Tang XR, Sun Y (2015). MiR-34c suppresses tumor growth and metastasis in nasopharyngeal carcinoma by targeting MET. Cell Death Dis.

[20] Xu YF, Mao YP, Li YQ, Ren XY, He QM, Tang XR (2015). MicroRNA-93 promotes cell growth and invasion in nasopharyngeal carcinoma by targeting Disabled homolog-2. Cancer Lett.

[21] Yi C, Wang Q, Wang L, Huang Y, Li L, Liu L (2012). MiR-663, a microRNA targeting p21(WAF1/CIP1), promotes the proliferation and tumorigenesis of nasopharyngeal carcinoma. Oncogene.

[22] Zaidi SK, Dowdy CR, van Wijnen AJ, Lian JB, Raza A, Stein JL (2009). Altered Runx1 subnuclear targeting enhances myeloid cell proliferation and blocks differentiation by activating a miR-24/MKP-7/ MAP kinase network. Cancer Res.

[23] Chen LC, Zhang AL, Li YL, Zhang K, Han L, Du W (2013). MiR-24 regulates the proliferation and invasion of glioma by ST7L via β-catenin/Tcf-4 signaling. Cancer Lett.

[24] Mishra PJ, Song B, Mishra PJ, Wang Y, Humeniuk R, Banerjee D (2009). MiR-24 tumor suppressor activity is regulated independent of p53 and through a target site polymorphism. PLoS ONE.

[25] Duan YT, Hu L, Liu B, Yu B, Li J, Yan M, et al. Tumor suppressor miR-24 restrains gastric cancer progression by downregulating RegIV. Mol Cancer. 2014;13:127. 10.1186/1476-4598-13-127 PMC4041902 24886316

[26] Fang Z, Tang J, Bai Y, Lin H, You H, Jin H (2015). Plasma levels of microRNA-24, microRNA-320a, and microRNA-423-5p are potential biomarkers for colorectal carcinoma. J Exp Clin Cancer Res.

[27] Organista-Nava J, Gomez-Gomez Y, IIIades-Aguiar B, Del Carmen Alarcón-Romero L, Saavedra-Herrera MV, Rivera-Ramírez AB, et al. High miR-24 expression is accociated with risk of relapse and poor survival in acute leukemia. Oncol Rep. 2015;33:1639–49. 10.3892/or.2015.3787 PMC4358084 25672522

[28] Meng FL, Wang W, Jia WD (2014). Diagnostic and prognostic significance of serum miR-24-3p in HBV-related hepatocellular carcinoma. Med Oncol.

[29] Wang S, Zhang R, Claret FX, Yang H (2014). Involvement of microRNA-24 and DNA methylation in resistance of nasopharyngeal carcinoma to ionizing radiation. Mol Cancer Ther.

[30] Srivastava N, Manvati S, Srivastava A, Pal R, Kalaiarasan P, Chattopadhyay S (2011). miR-24-2 controls H2AFX expression regardless of gene copy number alteration and induces apoptosis by targeting antiaopototic gene BCL-2: a potential for therapeutic intervention. Breast Cancer Res.

[31] Papadimitriou E, Vasilaki E, Vorvis C, Iliopoulos D, Moustakas A, Kardassis D (2012). Differential regulation of the two RhoA-specific GEF isoforms Net1/Net1A by TGF-β and miR-24: role in epithelial-to mesenchymal transition. Oncogene.

[32] Rodriguez-Pinilla SM, Sarrio D, Honrado E, Hardisson D, Calero F, Benitez J (2006). Prognostic significance of basal-like phenotype and fascin expression in node-negative invasive breast carcinomas. Clin Cancer Res.

[33] Vignjevic D, Schoumacher M, Gavert N, Janssen KP, Jih G, Laé M (2007). Fascin, a novel target of beta-catenin-TCF signaling, is expressed at the invasive front of human colon cancer. Cancer Res.

[34] Jawhari AU, Buda A, Jenkins M, Shehzad K, Sarraf C, Noda M (2003). Fascin, an actin-bunding protein, modulates colonic epithelial cell invasiveness and differentiation in vitro. Am J Pathol.

[35] Wu D, Chen L, Liao W, Ding Y, Zhang Q, Li Z (2010). Fascin1 expression predicts poor prognosis in ptiants with nasopharyngeal carcinoma and correlates with tumor invasion. Ann Oncol.

